# Supplementary material for: Metabolomic Profiling and Biological Investigation of the Marine Sponge-Derived Fungus Aspergillus sp. SYPUF29 in Response to NO Condition
Source: J Fungi (Basel). 2024 Sep 5;10(9):636. doi: 10.3390/jof10090636 (PMC11433098; doi:10.3390/jof10090636)
Supplement: Supplementary file 1 [file jof-10-00636-s001.zip › jof-3155118-supplementary.pdf]

Supporting Information

Metabolomic Profiling and Biological Investigation of the Marine Sponge-Derived Fungus *Aspergillus* sp. SYPUF29 in Response to NO Condition

Jiao Xiao [1,*], Xiuping Lin [2], Yanqiu Yang [3], Yingshu Yu [1], Yinyin Li [1], Mengjie Xu [4] and Yonghong Liu [1,*]

[1] Wuya College of Innovation, Shenyang Pharmaceutical University, Shenyang 110016, China
[2] Guangdong Key Laboratory of Marine Materia Medica, South China Sea Institute of Oceanology, Chinese Academy of Sciences, Guangzhou 510301, China
[3] College of Information Science and Engineering, Northeastern University, Shenyang 110819, China
[4] Department of Biological Sciences, XinZhou Normal University, Xinzhou 034000, China
* Correspondence: xj110121@126.com (J.X.); yonghongliu@scsio.ac.cn (Y.L.)

List of Supporting Information

The ITS gene sequence data of *Aspergillus* sp. SYPUF29

Figure S1: The Structures of differential compounds in AS1-AC-POS;

Figure S2: The Structures of differential compounds in AL3-AC-POS;

Figure S3: The Structures of differential compounds in AS1-AC-NEG;

Figure S4: The Structures of differential compounds in AL3-AC-NEG;

Figure S5: The BPC and EIC spectura of compounds SF1-SF4, SF6-SF8;

Figure S6: HRESIMS spectrum of SF1;

Figure S7: ^{1}H NMR (600 MHz, CD_3OD) spectrum of SF1;

Figure S8: ^{13}C NMR (150 MHz, CD_3OD) spectrum of SF1;

Figure S9: HRESIMS spectrum of SF2;

Figure S10: ^{1}H NMR (600 MHz, CD_3OD) spectrum of SF2;

Figure S11: ^{13}C NMR (150 MHz, CD_3OD) spectrum of SF2;

Figure S12: HRESIMS spectrum of SF3;

Figure S13: ^{1}H NMR (600 MHz, CD_3OD) spectrum o SF3;

Figure S14: ^{13}C NMR (150 MHz, CD_3OD) spectrum of SF3;

Figure S15: HRESIMS spectrum of SF4;

Figure S16: ^{1}H NMR (600 MHz, CD_3OD) spectrum of SF4;

Figure S17: ^{13}C NMR (150 MHz, CD_3OD) spectrum of SF4;

Figure S18: ^{1}H NMR (600 MHz, CD_3OD) spectrum of SF5;

Figure S19: ^{13}C NMR (150 MHz, CD_3OD) spectrum of SF5;

Figure S20: HRESIMS spectrum of SF6;

Figure S21: ^{1}H NMR (600 MHz, CD_3OD) spectrum of SF6;

Figure S22: ^{13}C NMR (150 MHz, CD_3OD) spectrum of SF6;

Figure S23: HRESIMS spectrum of SF7;

Figure S24: ^{1}H NMR (600 MHz, CD_3OD) spectrum of SF7;

Figure S25: ^{13}C NMR (150 MHz, CD_3OD) spectrum of SF7;

Figure S26: HRESIMS spectrum of SF8;

Figure S27: ^{1}H NMR (600 MHz, CD_3OD) spectrum of SF8;

Figure S28: ^{13}C NMR (150 MHz, CD_3OD) spectrum of SF8;

Figure S29: ^{1}H NMR (600 MHz, $CDCl_3$) spectrum of SF9;

Figure S30: ^{13}C NMR (150 MHz, $CDCl_3$) spectrum of SF9;

Table S1: ^{1}H NMR and ^{13}C NMR data of compounds SF1 in $CDCl_3$ and SF2 in CD_3OD;

Table S2: ^{1}H NMR and ^{13}C NMR data of compounds SF3 in CD_3OD;

Table S3: ^{1}H NMR and ^{13}C NMR data of compounds SF4 and SF5 in CD_3OD;

Table S4: ^{1}H NMR and ^{13}C NMR data of compounds SF6 in CD_3OD;

Table S5: ^{1}H NMR and ^{13}C NMR data of compounds SF7 in CD_3OD;

List of Supporting Information

The ITS gene sequence data of *Aspergillus* sp. SYPUF29

ACCTGCGGAAGGATCATTACCGAGTGCTGGGTCCTTCGGGGCCCAAC-
CTCCCACCCGTGCTTACCGTACCCTGTTGCTTCGGCGGGCCCGCCTTCGGGCGGCCCGGGGCCTGCCCCCGGG
ACCGCGCCCGCCGGAGACCCCAATGGAACACTGTCTGAAAGCGTGCAGTCTGAGTCGATTGA-
TACCAATCAGTCAAAACTTTCAACAATGGATCTCTTGGTTCCGGCATCGATGAAGAACGCAGCGAAATGCGAT
AACTAATGTGAATTGCAGAATTCAGTGAATCATCGAGTCTTTGAACGCACATT-
GCGCCCCCTGGTATTCCGGGGGGCATGCCTGTCCGAGCGTCATTTCTCCCCTCCAGCCCCGCTGGTTGTTGGGC
CGCGCCCCCCCGGGGGCGGGCCTCGAGAGAAACGGCGGCACCGTCCGGTCCTCGAGCG-
TATGGGGCTCTGTCACCCGCTCTATGGGCCCGGCCGGGGCTTGCCTCGACCCCCAATCTTCTCAGATTGACCTC
GGATCAGGTAGGGATACCCGCTGAACTTAAGCATATCAAT

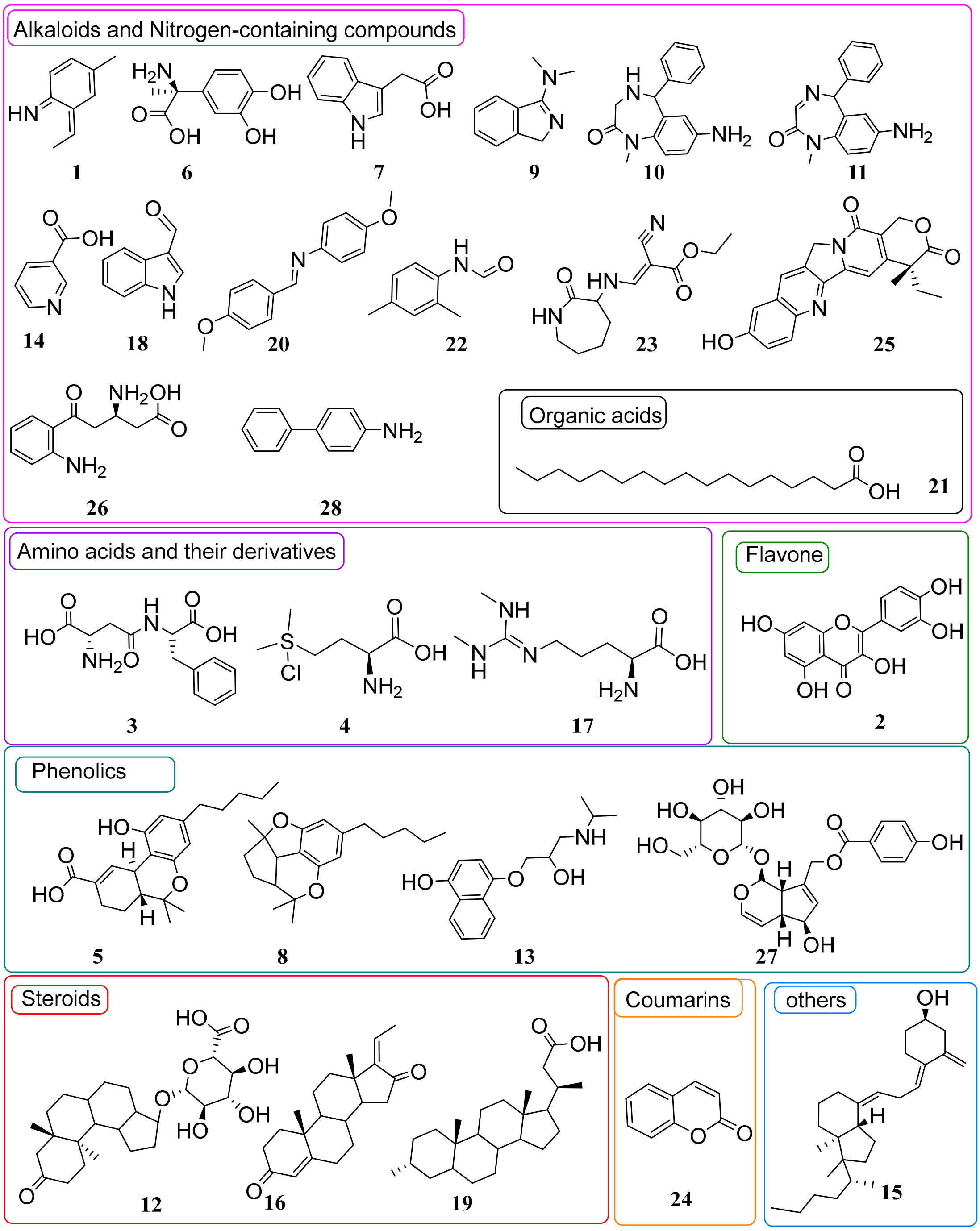

Figure S1. The Structures of differential compounds in AS1-AC-POS.

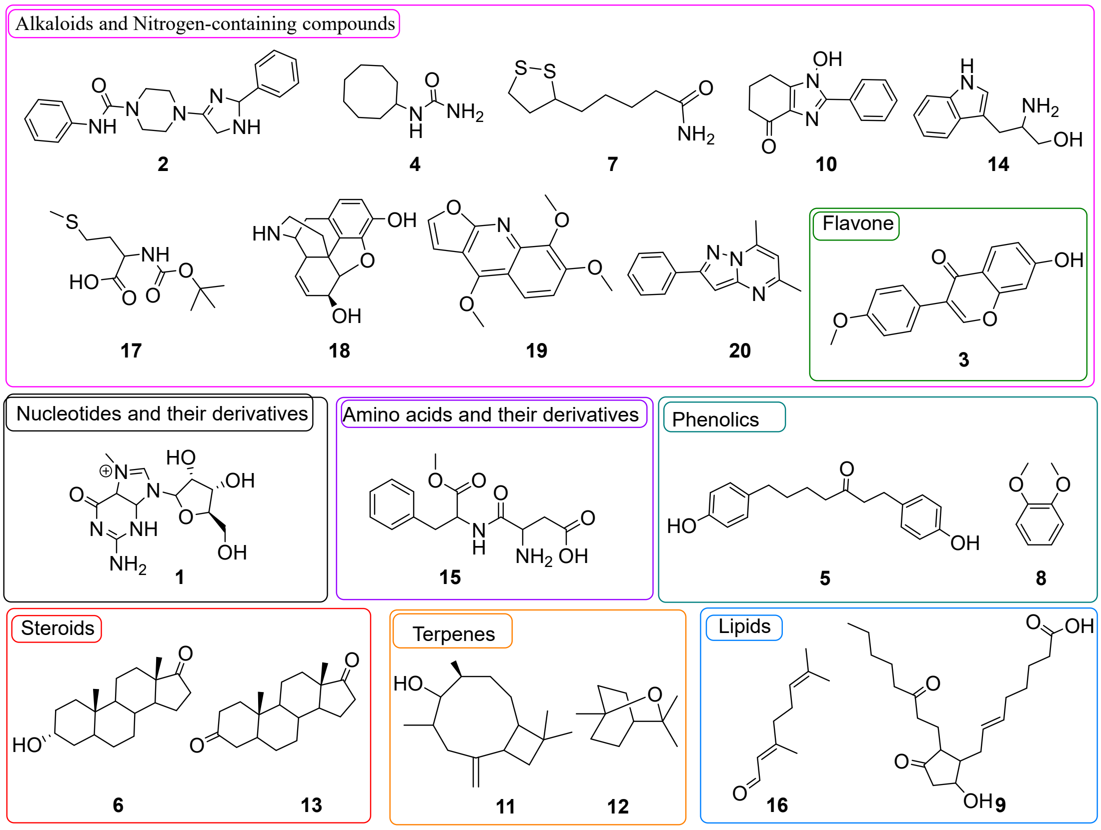

Figure S2. The Structures of differential compounds in AL3-AC-POS.

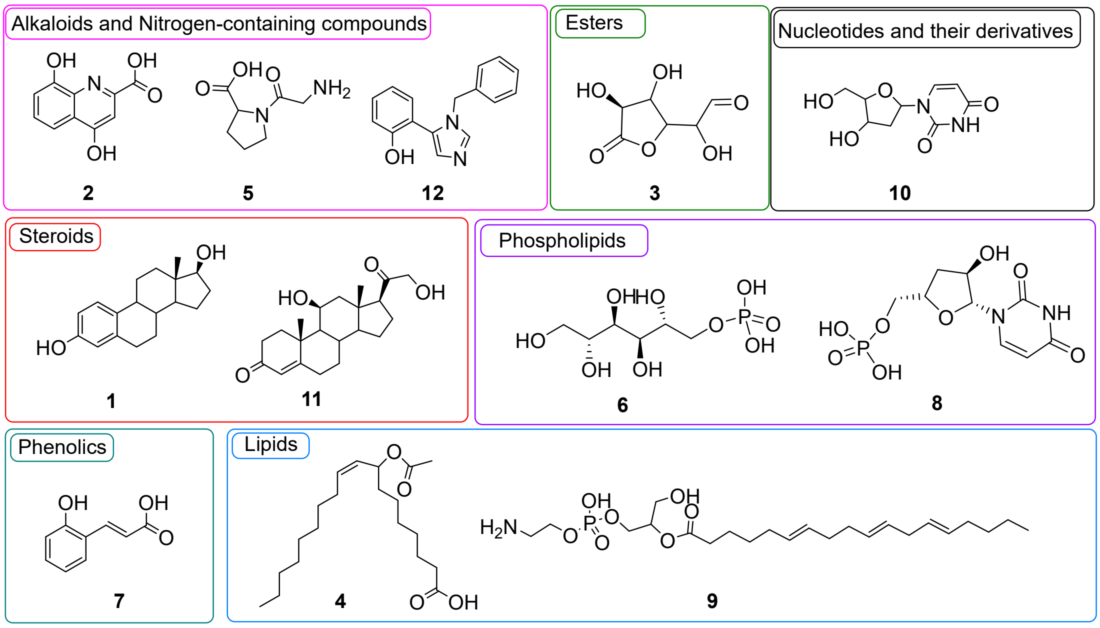

Figure S3. The Structures of differential compounds in AS1-AC-NEG.

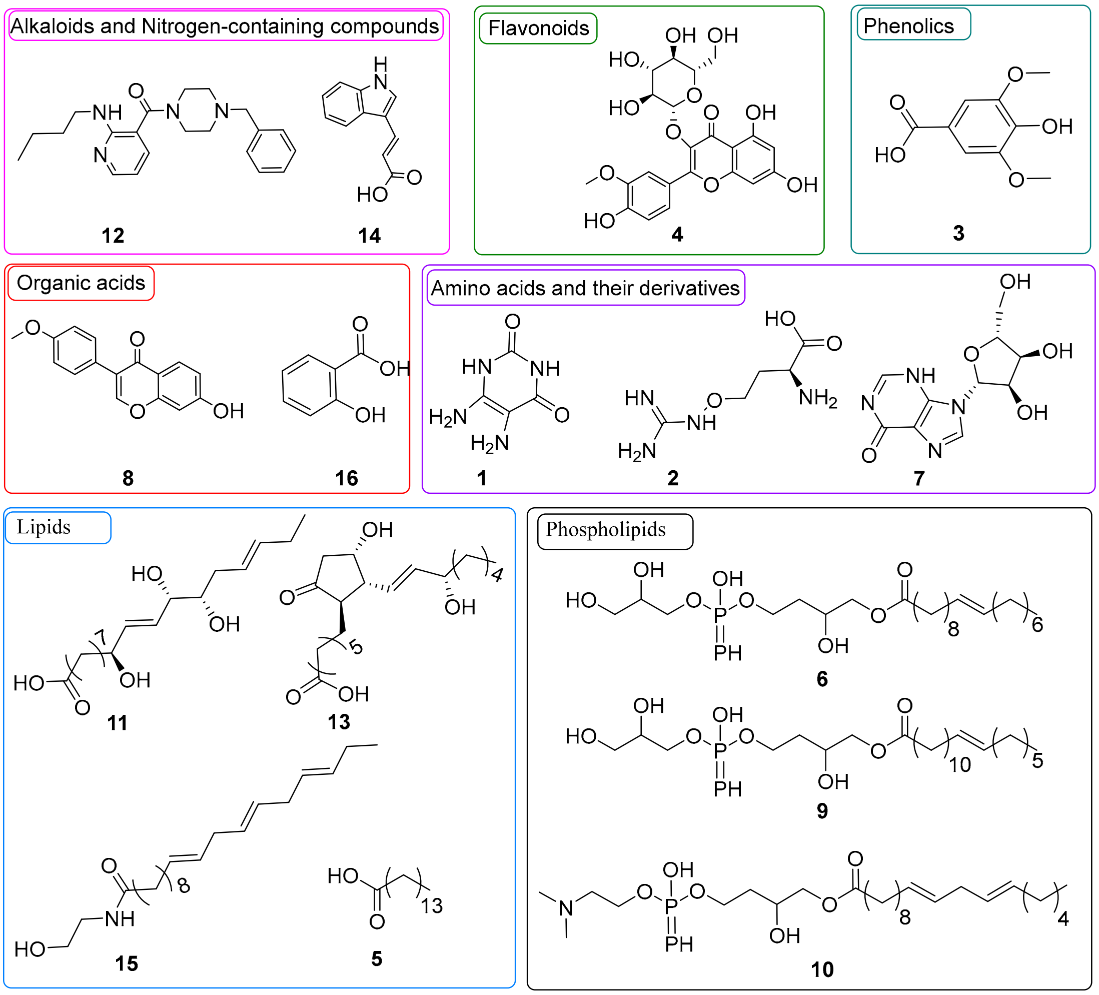

Figure S4. The Structures of differential compounds in AL3-AC-NEG.

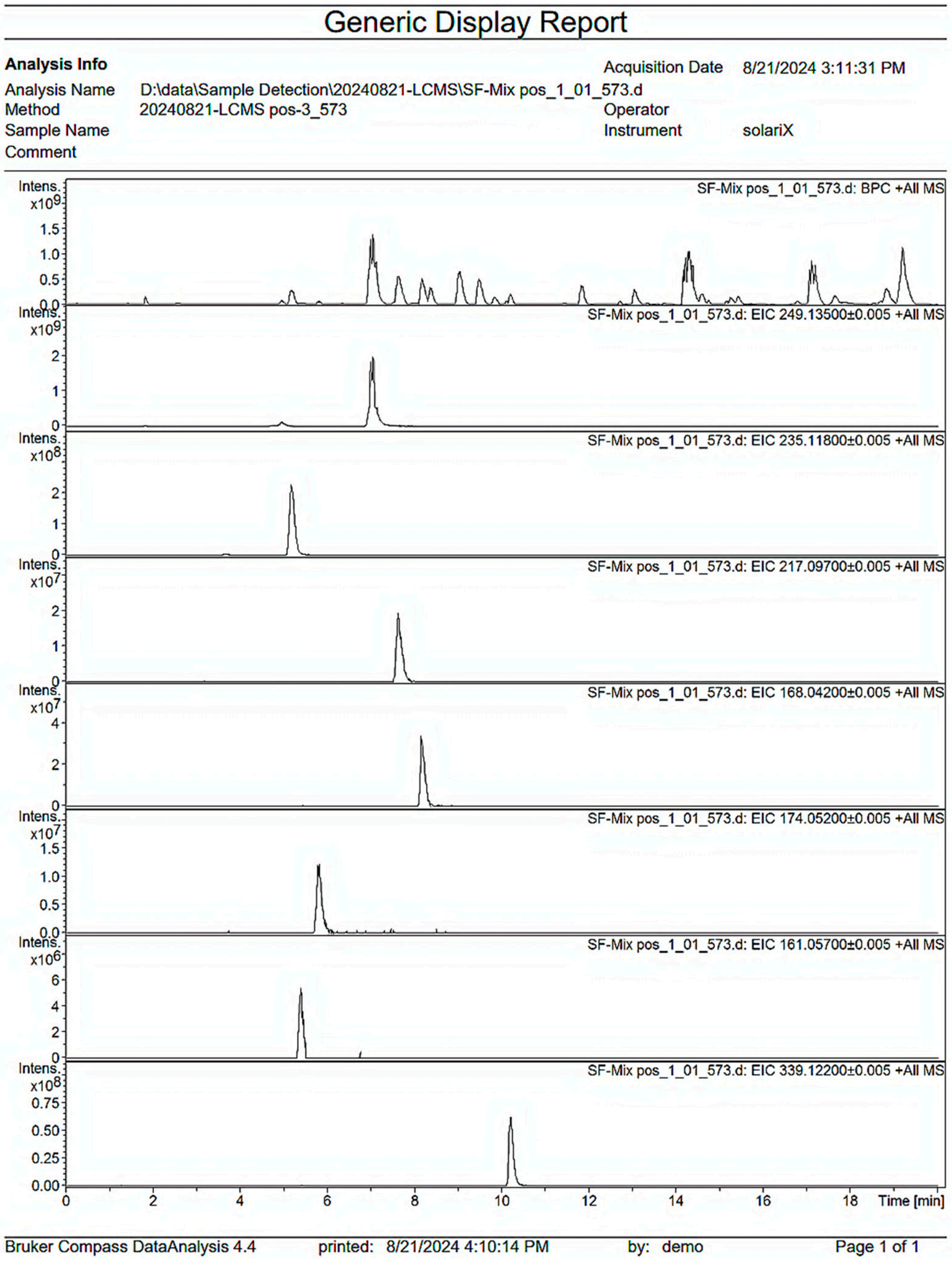

Figure S5. The BPC and EIC spectura of compounds **SF1-SF4**, **SF6-SF8.**

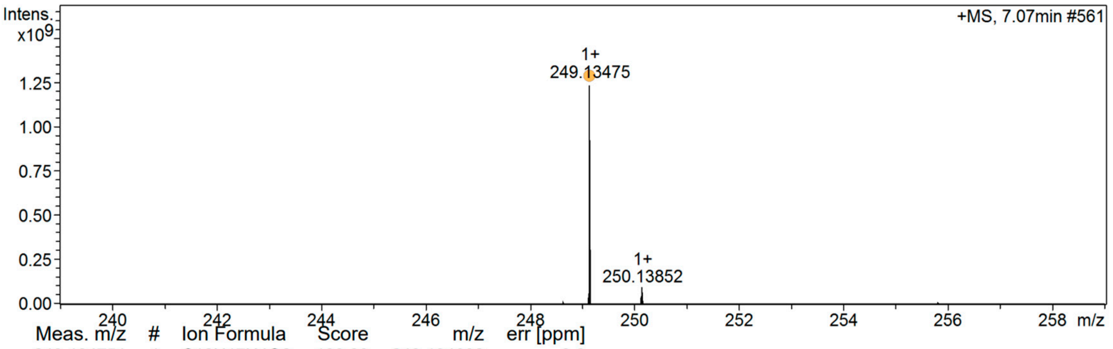

Meas. m/z	#	Ion Formula	Score	m/z	err [ppm]
249.134751	1	C12H17N4O2	100.00	249.134602	-0.6

Figure S6. HRESIMS spectrum of SF1.

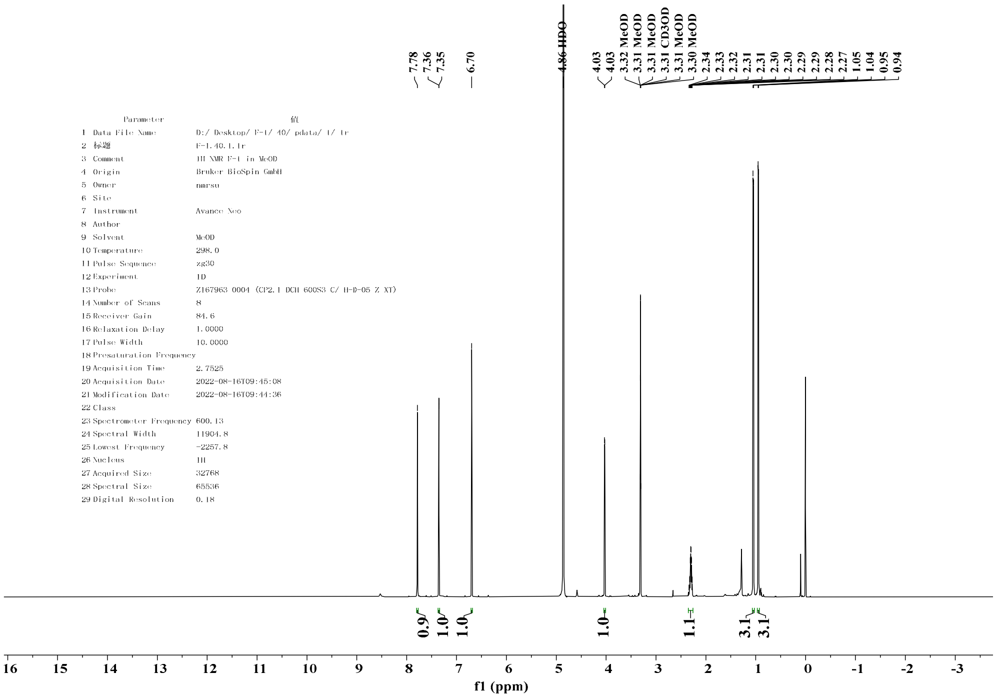

Figure S7. ^{1}H NMR (600 MHz, CD_3OD) spectrum of **SF1.**

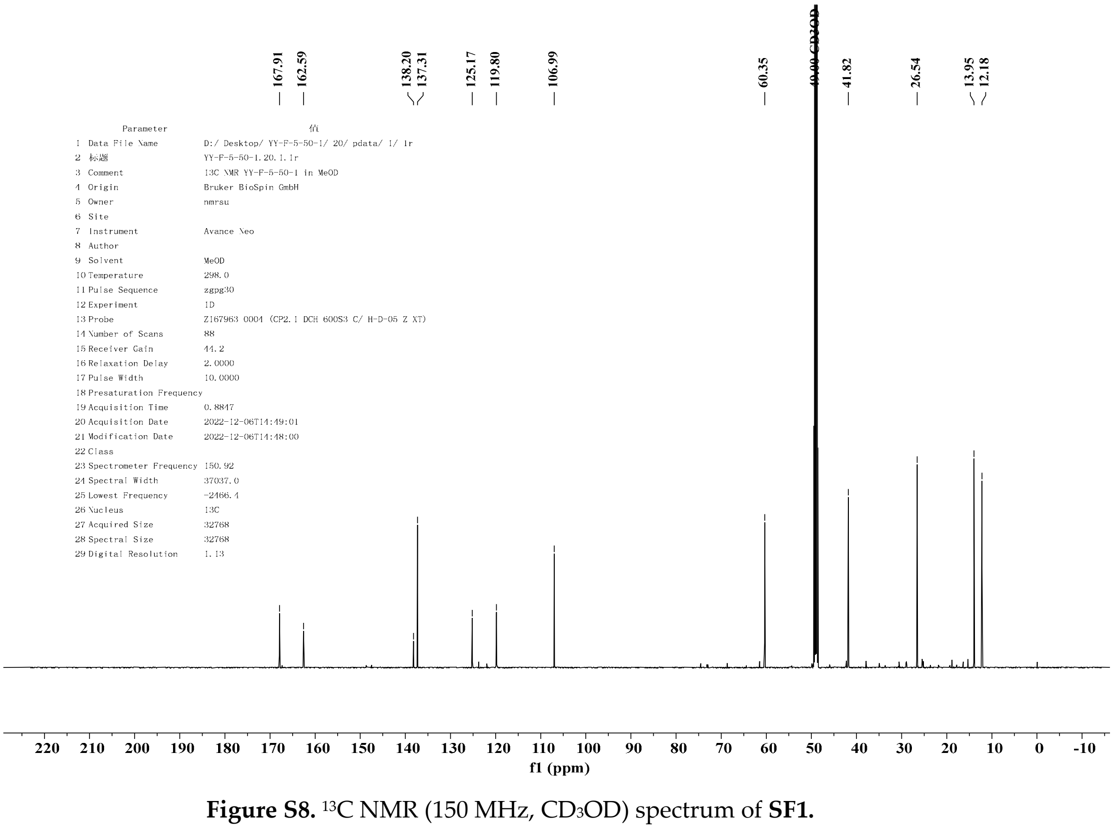

Figure S8. ^{13}C NMR (150 MHz, CD_3OD) spectrum of **SF1.**

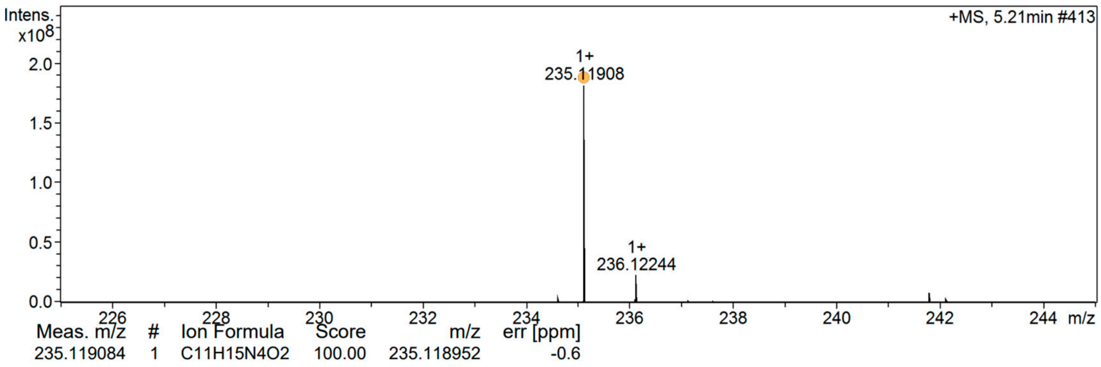

Figure S9. HRESIMS spectrum of **SF2.**

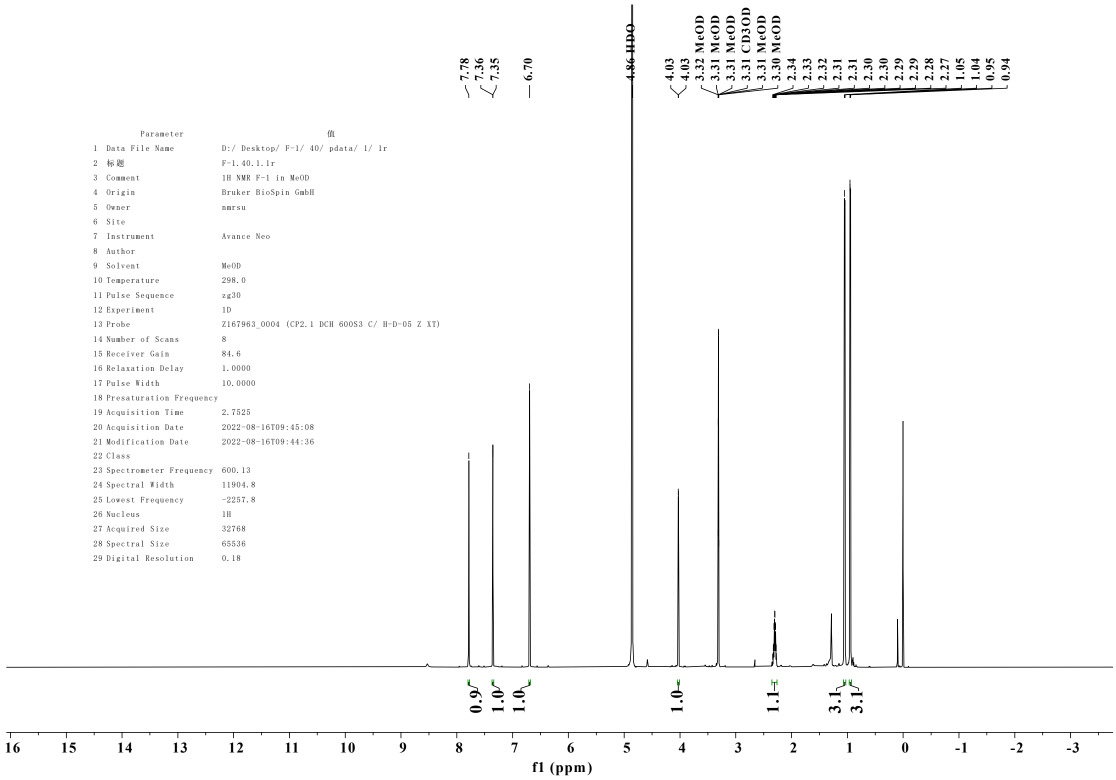

Figure S10. ^{1}H NMR (600 MHz, CD_3OD) spectrum of **SF2.**

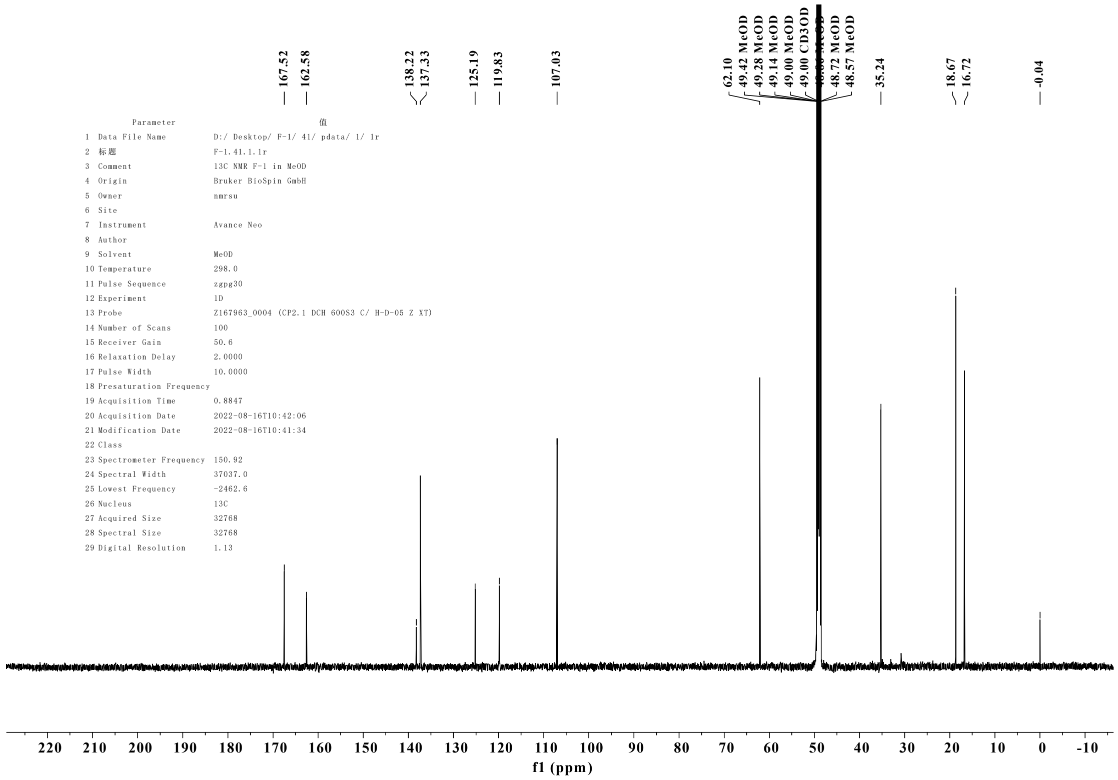

Figure S11. ^{13}C NMR (150 MHz, CD_3OD) spectrum of **SF2.**

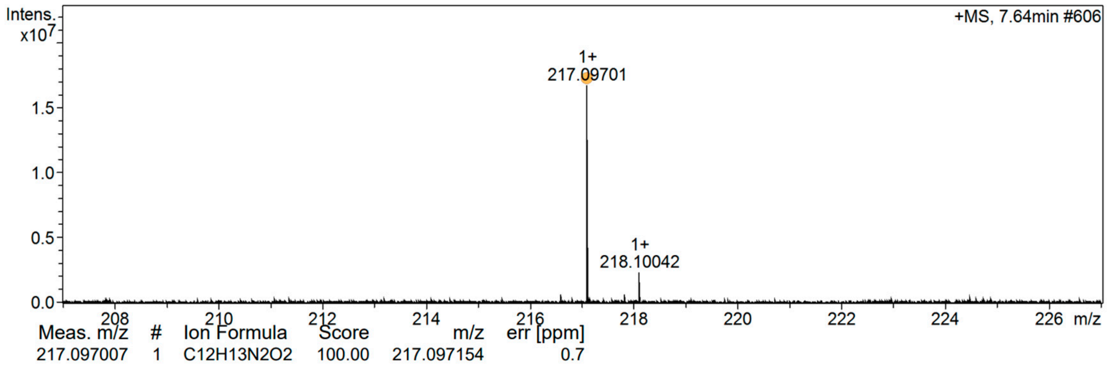

Figure S12. HRESIMS spectrum of **SF3.**

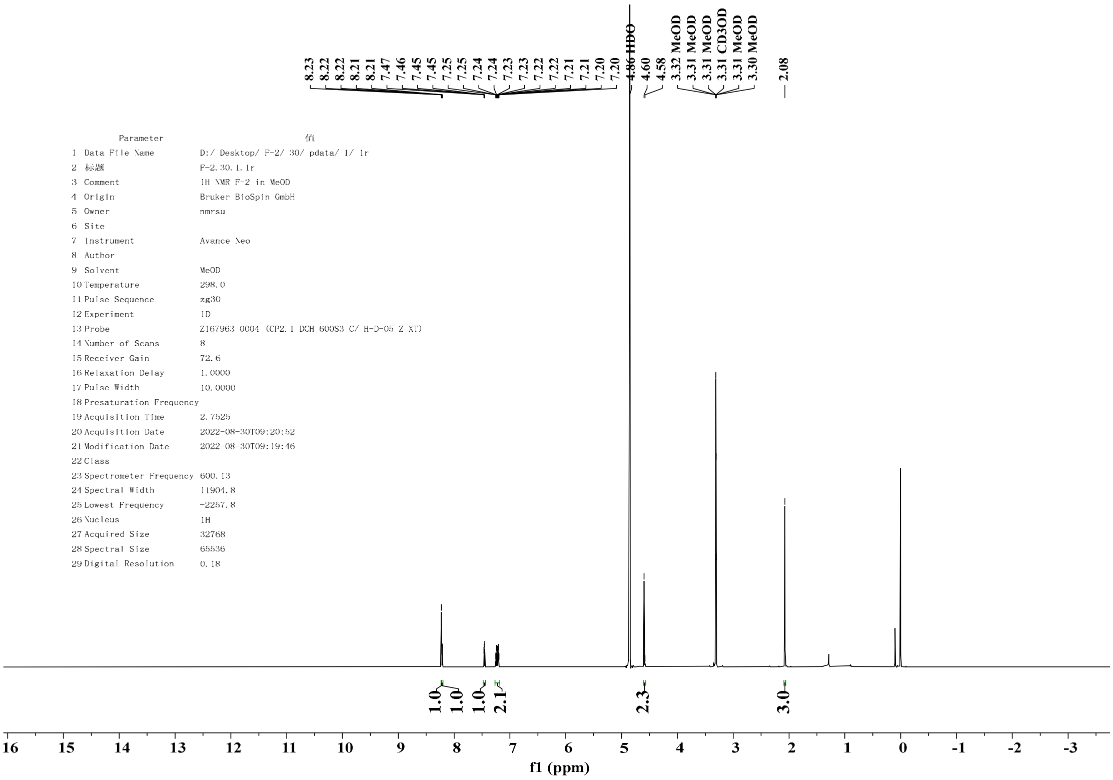

Figure S13. ^{1}H NMR (600 MHz, CD_3OD) spectrum o **SF3.**

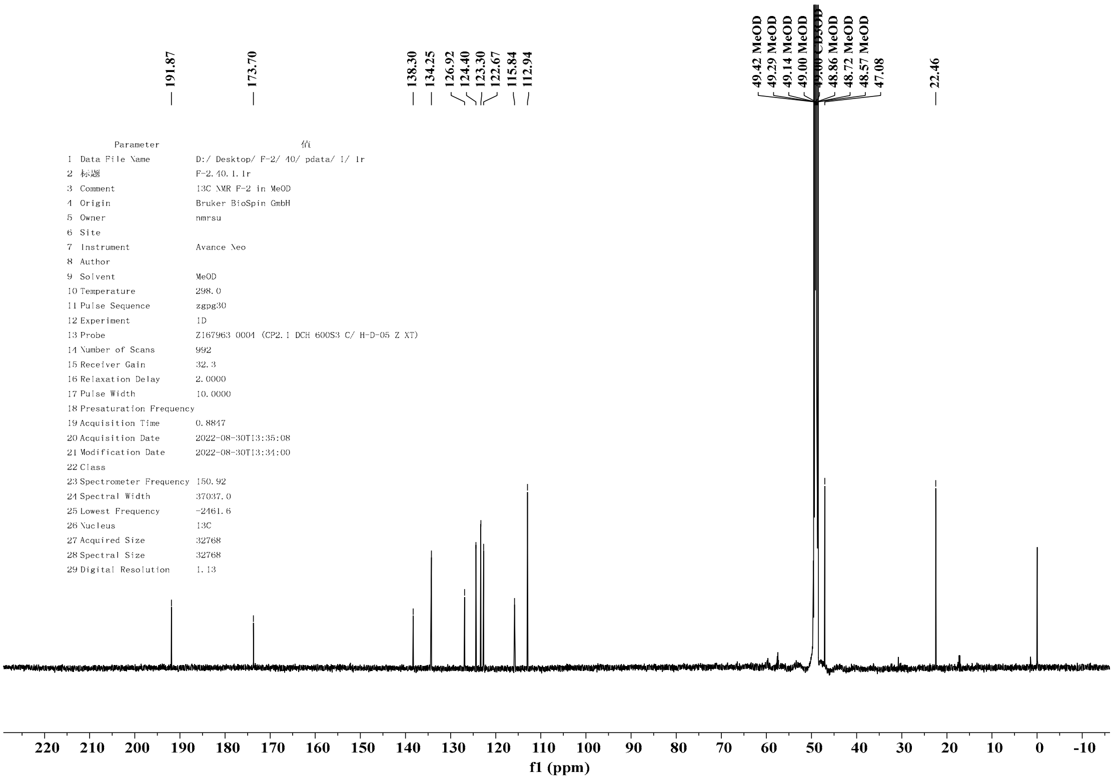

Figure S14. ^{13}C NMR (150 MHz, CD_3OD) spectrum of **SF3.**

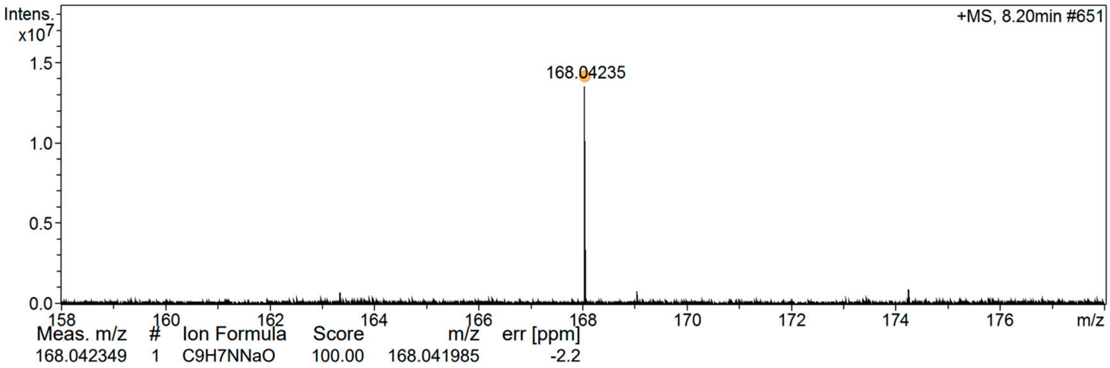

Figure S15. HRESIMS spectrum of **SF4.**

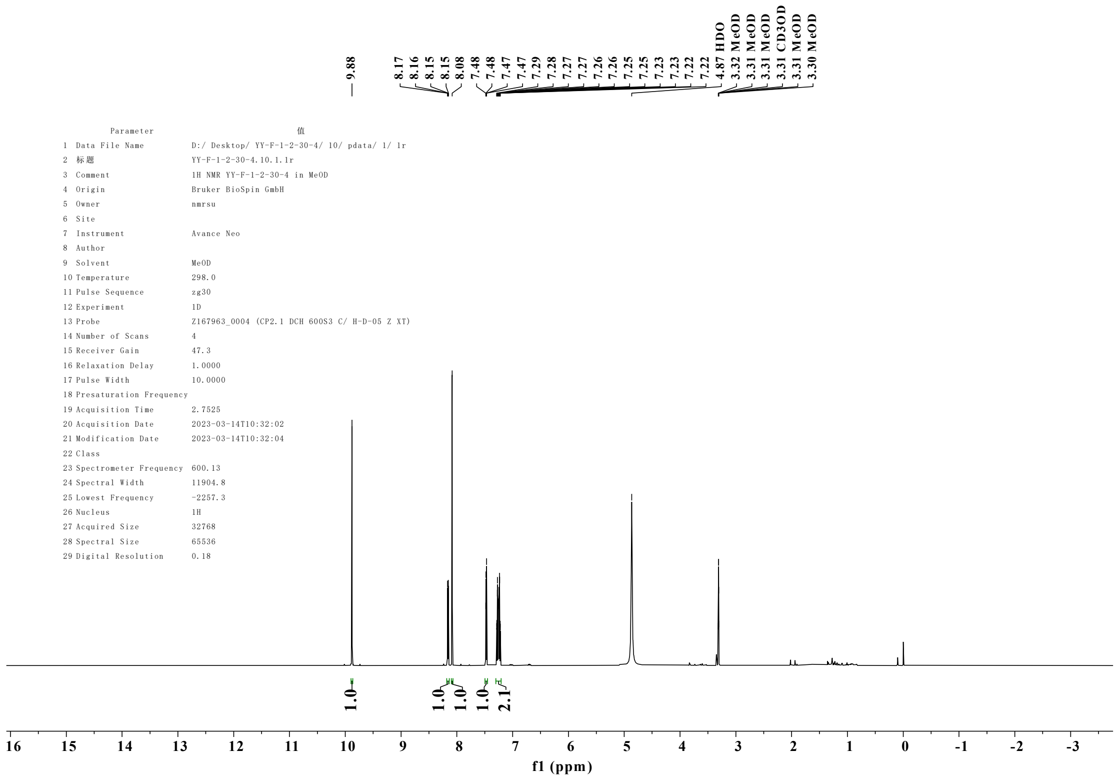

Figure S16. ^{1}H NMR (600 MHz, CD_3OD) spectrum of **SF4.**

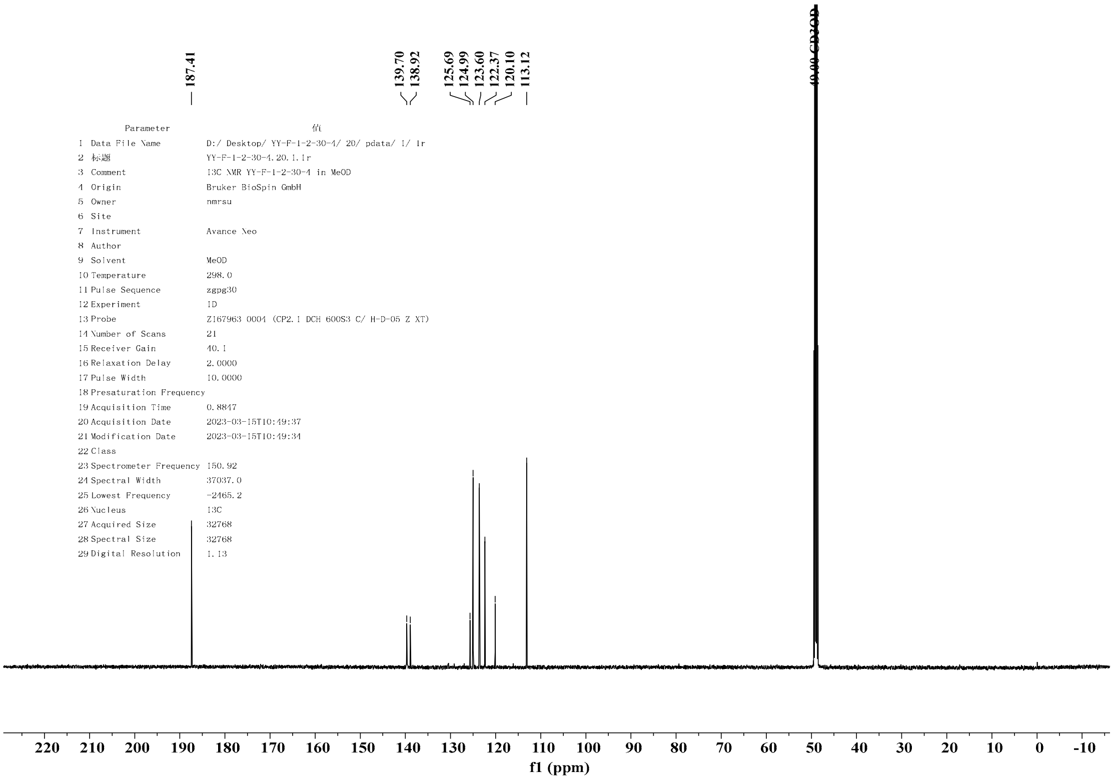

Figure S17. ^{13}C NMR (150 MHz, CD_3OD) spectrum of **SF4.**

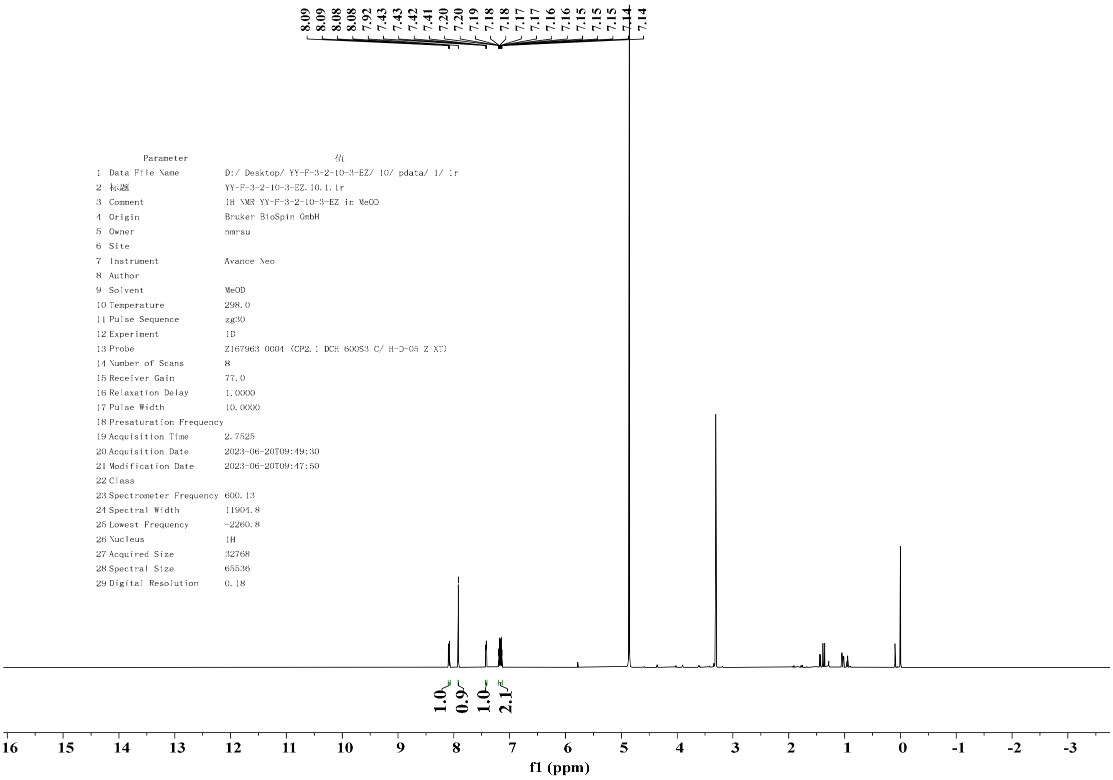

Figure S18. H NMR (600 MHz, CD_3OD) spectrum of **SF5.**

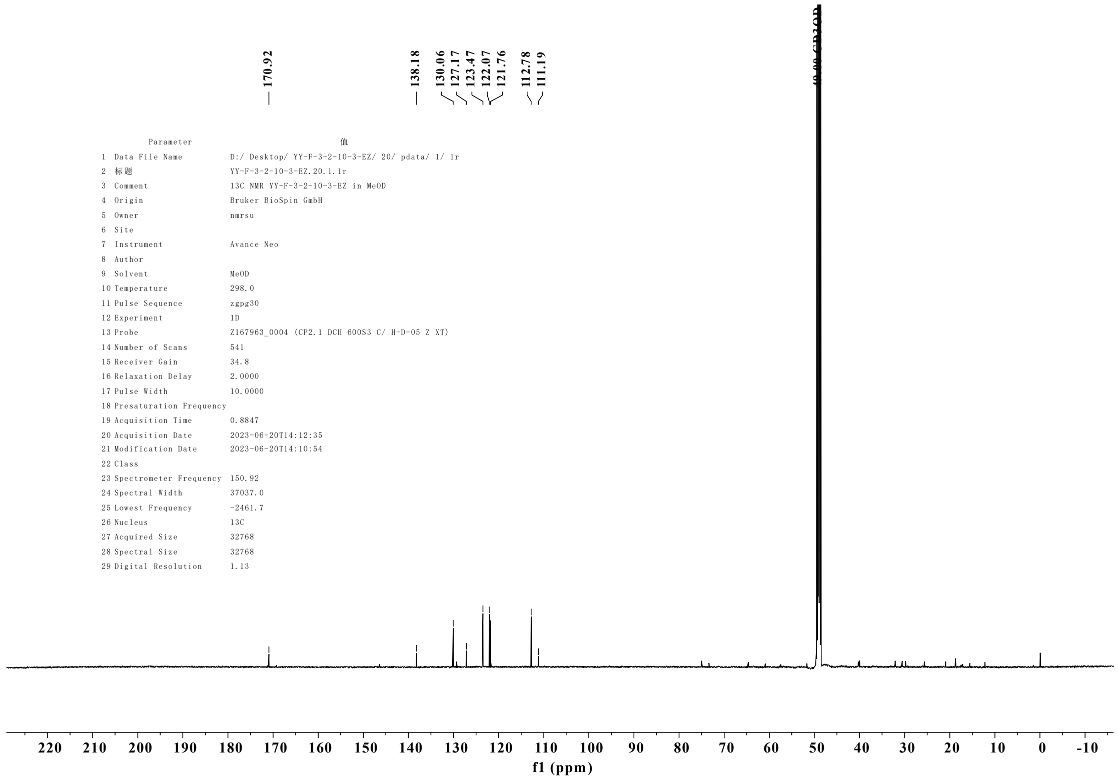

Figure S19. C NMR (150 MHz, CD3OD) spectrum of **SF5.**

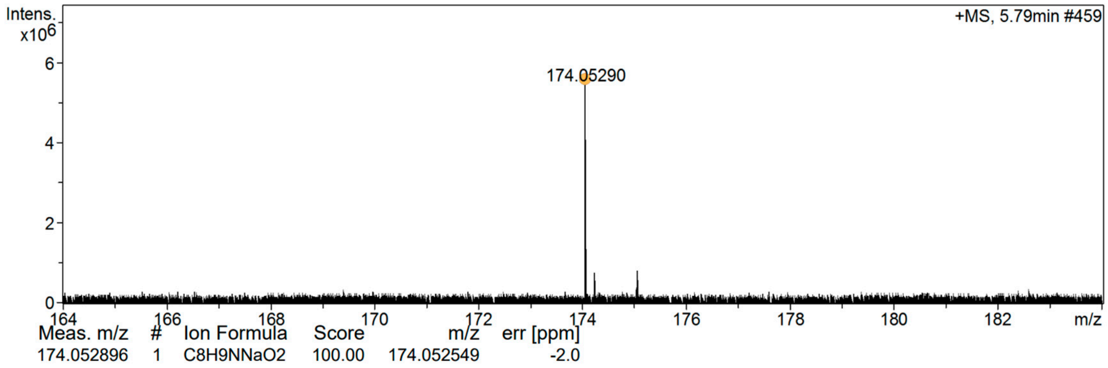

Figure S20. HRESIMS spectrum of **SF6.**

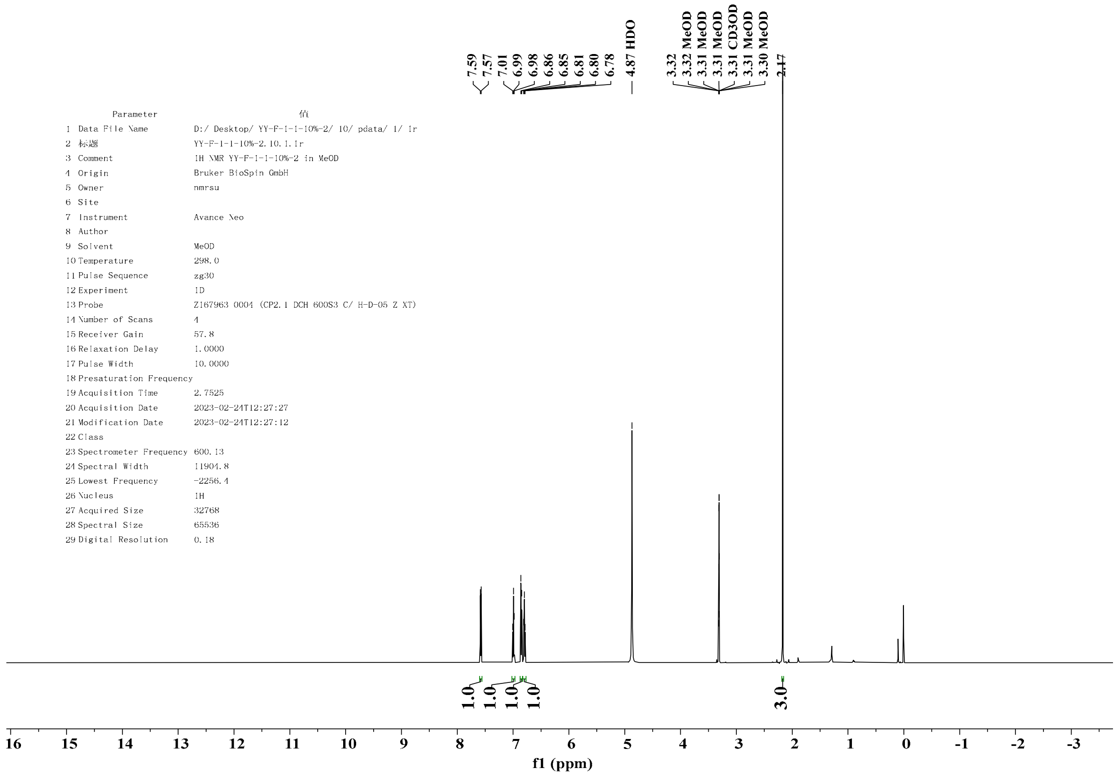

Figure S21. H NMR (600 MHz, CD_3OD) spectrum of **SF6.**

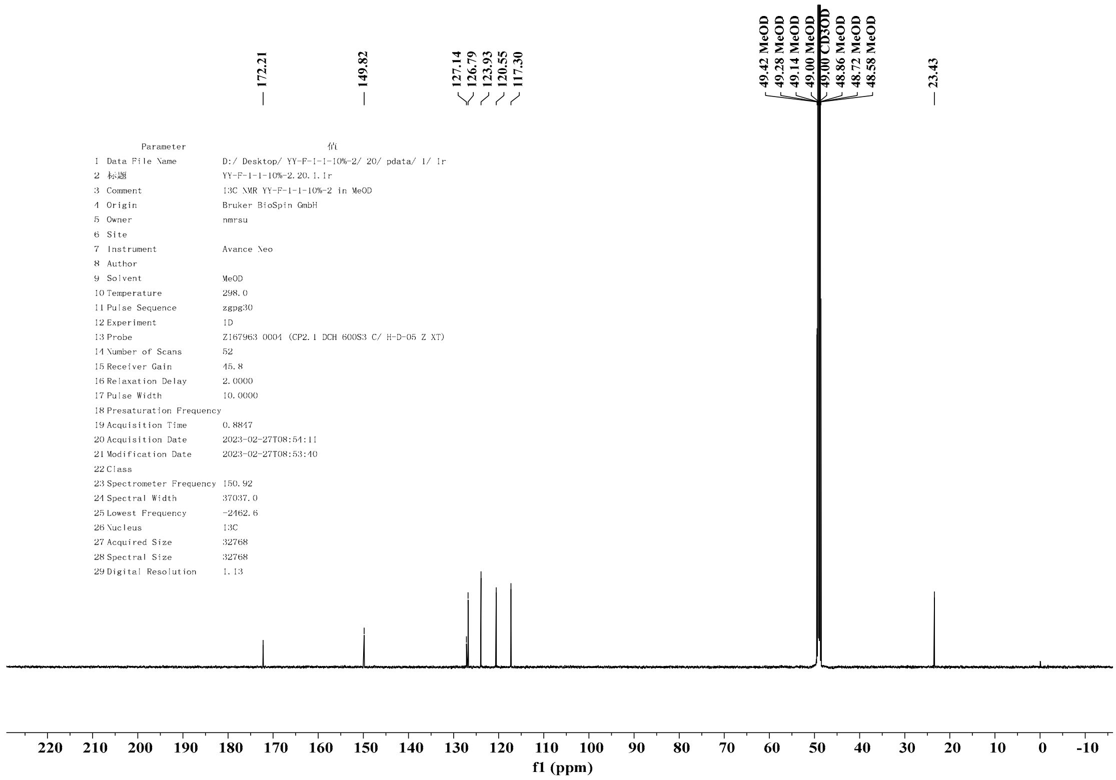

Figure S22. ^{13}C NMR (150 MHz, CD_3OD) spectrum of **SF6.**

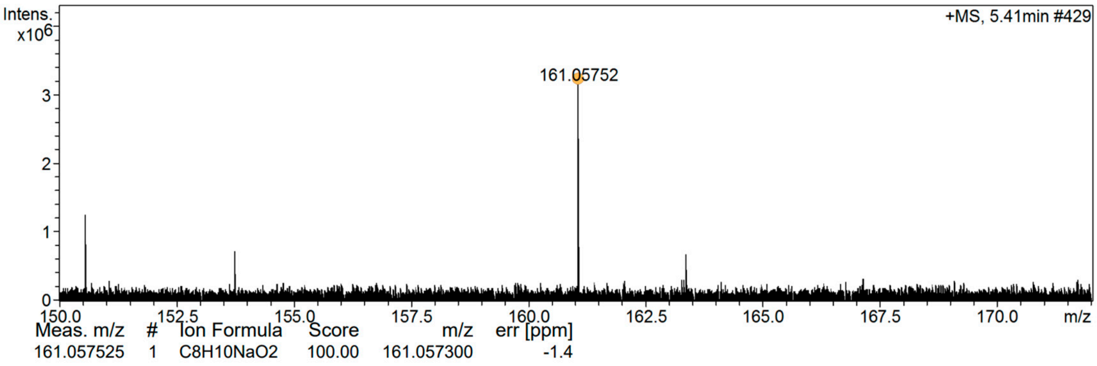

Figure S23. HRESIMS spectrum of **SF7.**

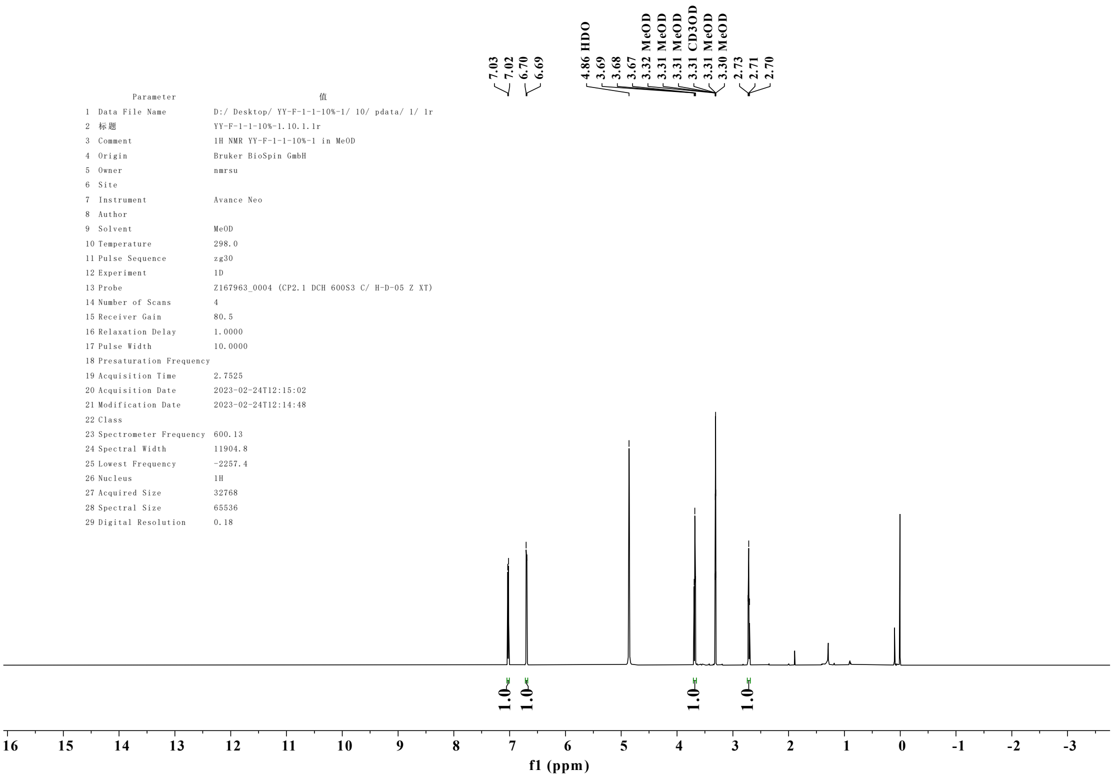

Figure S24. ^{1}H NMR (600 MHz, CD_3OD) spectrum of **SF7.**

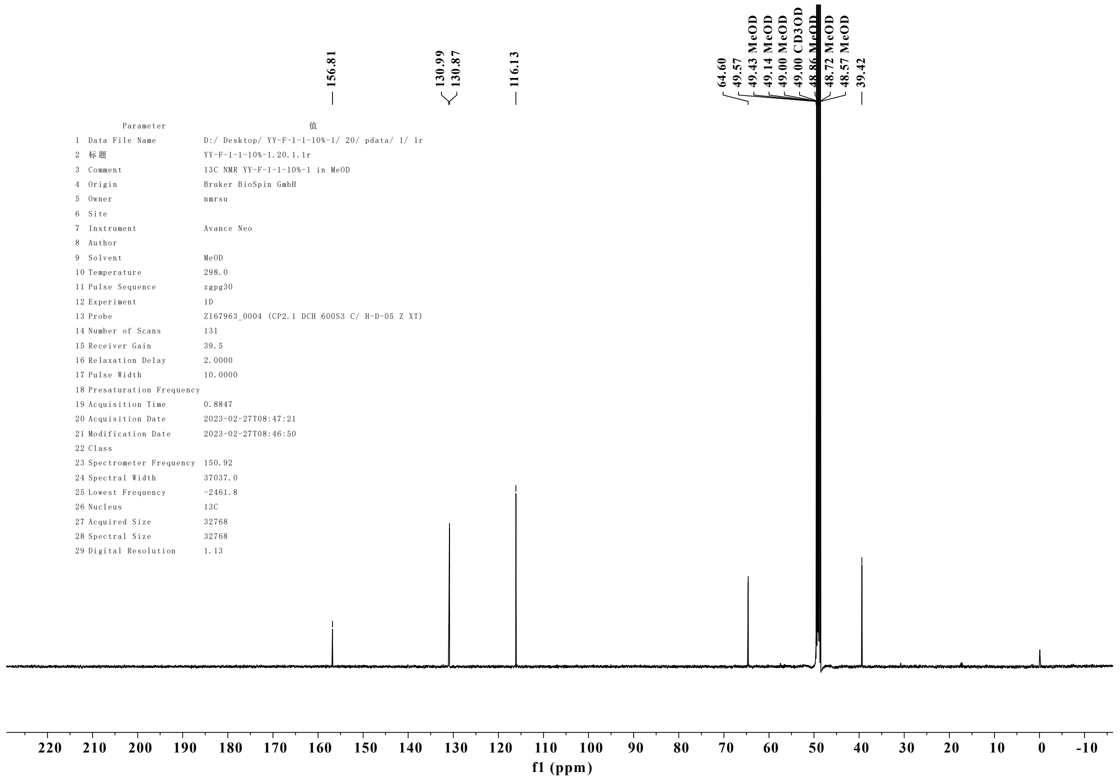

Figure S25. ^{13}C NMR (150 MHz, CD_3OD) spectrum of **SF7.**

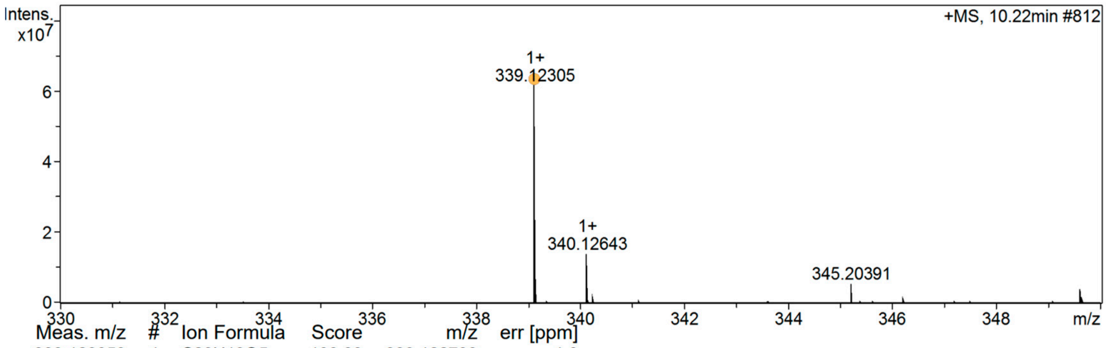

Meas. m/z	#	Ion Formula	Score	m/z	err [ppm]
339.123053	1	C20H19O5	100.00	339.122700	-1.0

Figure S26. HRESIMS spectrum of **SF8.**

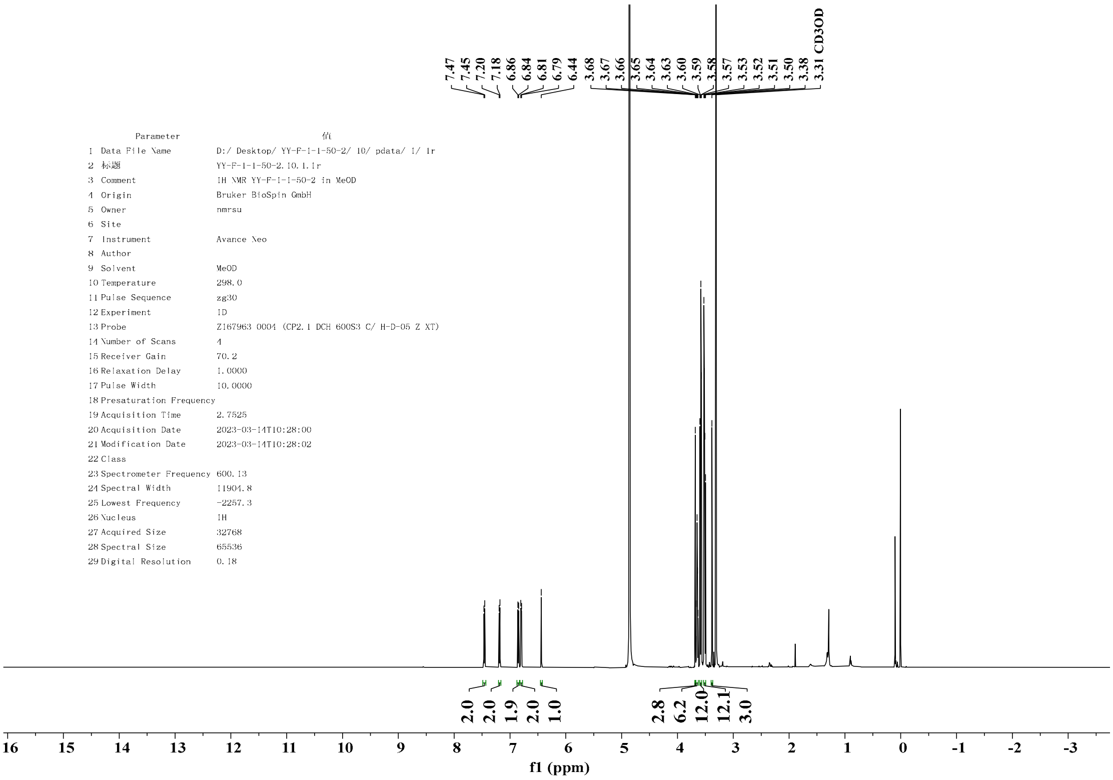

Figure S27. ^{1}H NMR (600 MHz, CD_3OD) spectrum of **SF8.**

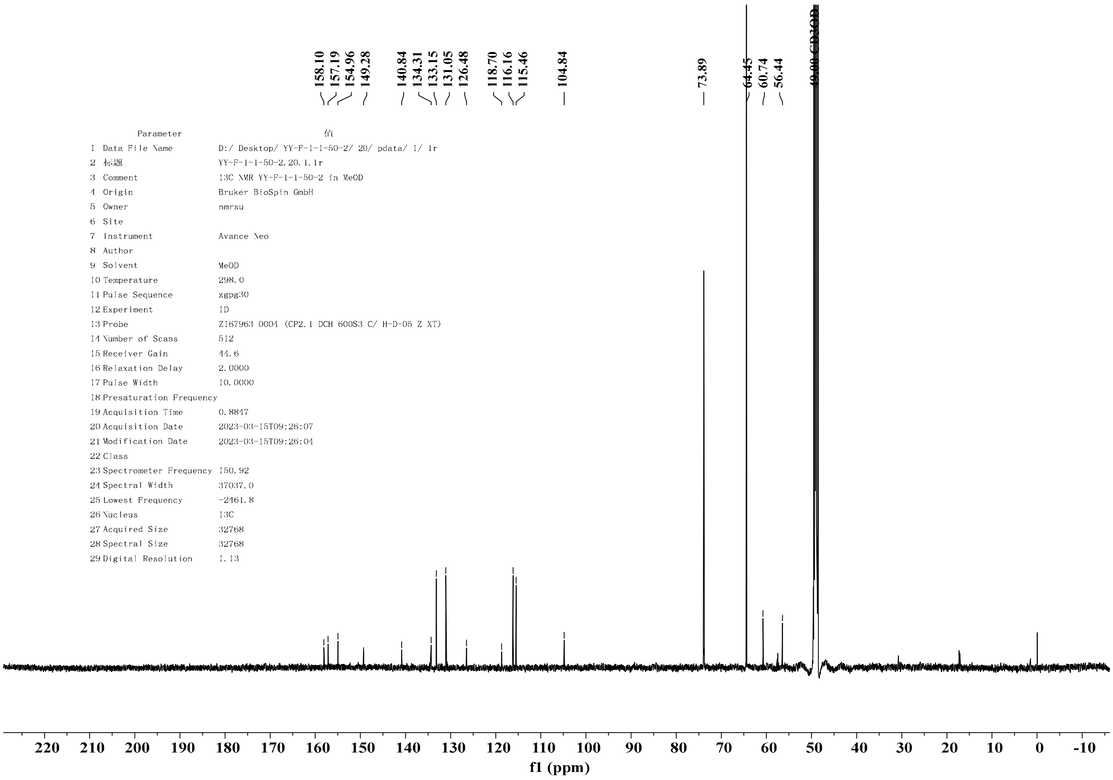

Figure S28. ^{13}C NMR (150 MHz, CD_3OD) spectrum of **SF8.**

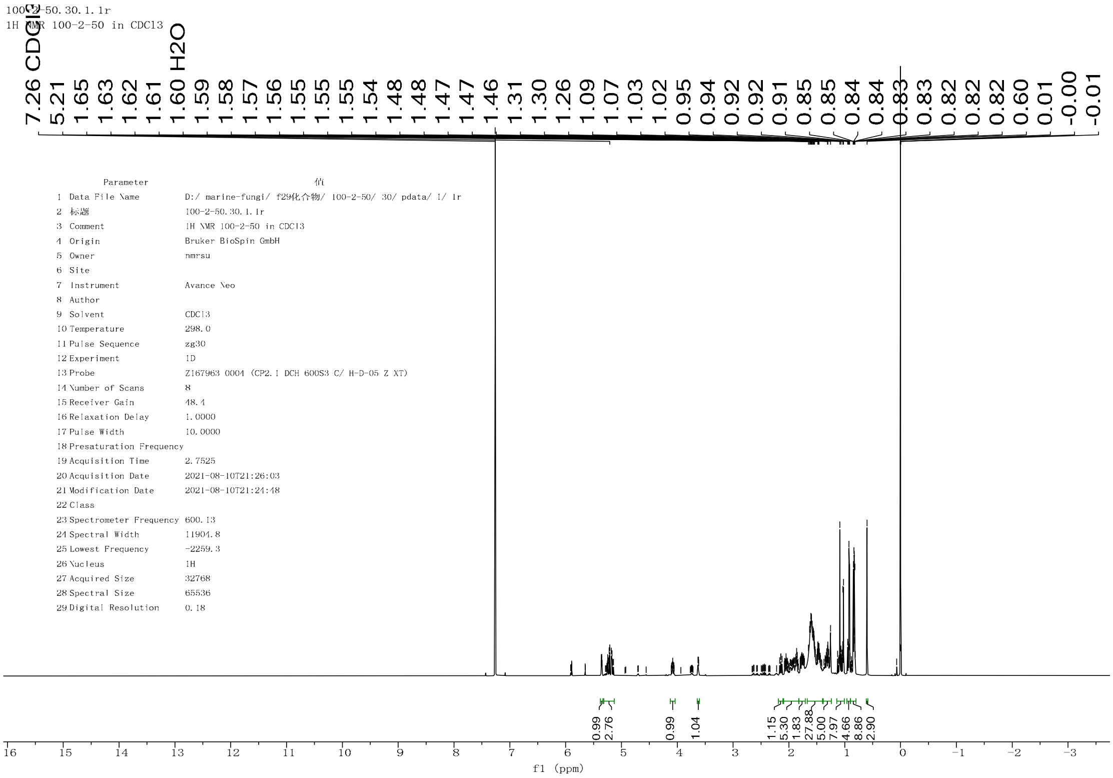

Figure S29. ^{1}H NMR (600 MHz, $CDCl_3$) spectrum of **SF9.**

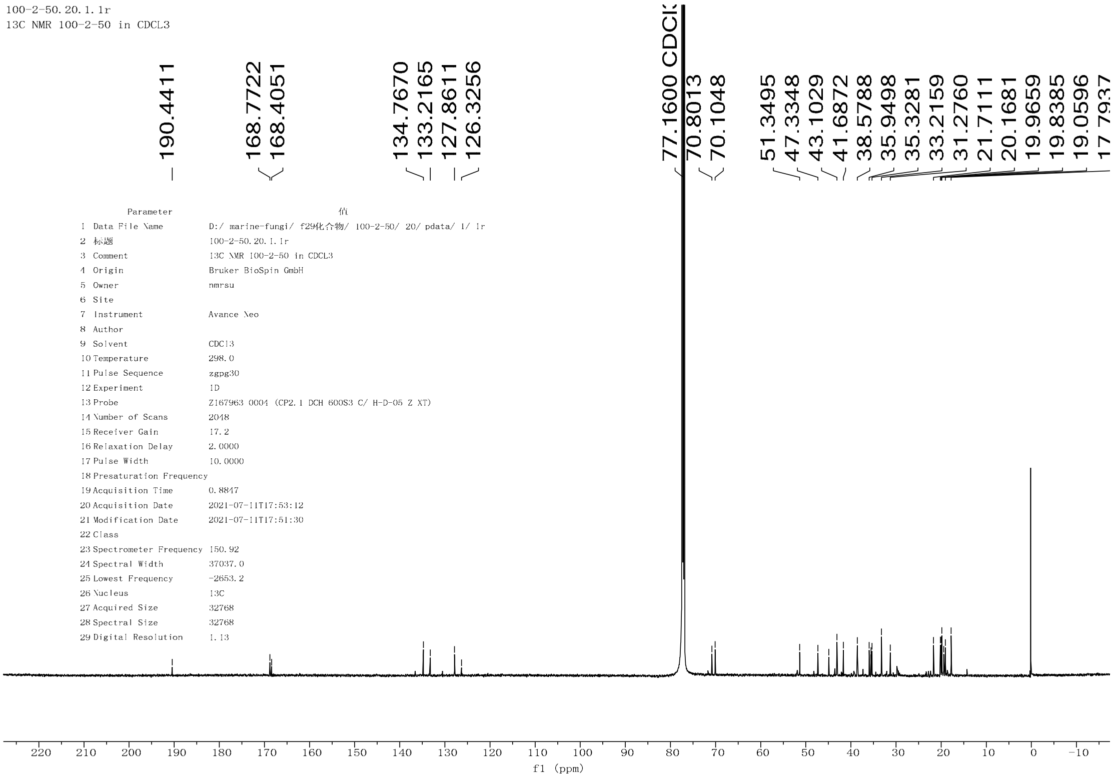

Figure S30. ^{13}C NMR (150 MHz, $CDCl_3$) spectrum of **SF9.**

Table S1. ^{1}H NMR and ^{13}C NMR data of compounds SF1 in $CDCl_3$ and SF2 in CD_3OD[1].

NO.	SF1		SF2	
	δ_H (*J* in Hz)	δ_C	δ_H (*J* in Hz)	δ_C
1		165.5		167.5
2	4.21 (d, 2.6)	59.6	4.04 (d, 3.1)	62.1
3	2.23 (m)	39.5	2.32 (m)	35.3
4	1.40 (m)	25.9	1.06 (d, 7.1)	18.7
5	0.94 (d, 6.9)	11.9	0.96 (d, 6.9)	16.7
6	0.99 (t, 7.4)	13.5		
1'		165.1		162.6
2'		125.0		125.2
3'	6.73 (s)	105.0	6.71 (s)	107.0
4'	7.19 (s)	137.7	7.37 (s)	138.2
5'	7.73 (s)	117.3	7.80 (s)	119.8
6'		135.3		137.3

The absolute configuration of compounds **SF1** ($[\alpha]_D^{20}$ +75.3, c 0.1, MeOH) and **SF2** (($[\alpha]_D^{20}$ +51.2, c 0.1)+51.2) were identified as 2*R*, 3*S*, based on the same positive optical rotation value with literature [1].

Table S2. ^{1}H NMR and ^{13}C NMR data of compounds SF3 in CD_3OD[2].

NO.	δ_H (*J* in Hz)	δ_C	NO.	δ_H (*J* in Hz)	δ_C
2	8.23 (s)	134.2	3a		126.9
3		115.8	7a		138.3
4	8.21 (m)	122.7	1'		191.9
5	7.23 (m)	123.3	2'	4.60 (s)	47.1
6		124.4	4'		173.7
7	7.46 (dd, 7.6, 1.4)	112.9	5'	2.08 (s)	22.5

Table S3. ^{1}H NMR and ^{13}C NMR data of compounds SF4 [3]and SF5[4] in CD_3OD.

NO.	SF4		SF5	
	δ_H (*J* in Hz)	δ_C	δ_H (*J* in Hz)	δ_C
2	8.08 (s)	139.7	7.92 (s)	130.1
3		120.1		123.5
4		123.6		127.2
5	8.16 (dd, 7.7, 1.2)	122.4	8.09 (dd, 8.0, 2.2)	112.8
6	7.25 (m)	125.0	7.17 (m)	122.1
7	7.26 (m)	113.1	7.17 (m)	121.8
8	7.47 (dd, 7.0, 2.0)	125.7	7.42 (dd, 8.0, 2.2)	111.2
9		138.9		138.2
10	9.88 (s)	187.4		170.9

Table S4. ^{1}H NMR and ^{13}C NMR data of compounds SF6[5] in CD_3OD.

NO.	δ_H (*J* in Hz)	δ_C	NO.	δ_H (*J* in Hz)	δ_C
1		127.1	5	6.80 (t, 7.6)	123.9
2		149.8	6	7.58 (d, 8.0)	120.5
3	6.85 (d, 8.0)	117.3	7		172.2
4	6.99 (t, 7.6)	126.8	8	2.17 (s)	23.4

Table S5. ^{1}H NMR and ^{13}C NMR data of compounds SF7[6] in CD_3OD.

NO.	δ_H (J in Hz)	δ_C	NO.	δ_H (J in Hz)	δ_C
1		156.8	6	6.70 (d, 8.4)	116.1
2	6.70 (d, 8.4)	116.1	7	3.68 (t, 7.2)	39.4
3	7.03 (d, 8.4)	130.9	8	2.71 (t, 7.2)	64.6
4		131.0	9		123.5
5	7.03 (d, 8.4)	130.9			

Table S6. ^{1}H NMR and ^{13}C NMR data of compounds SF8[7] in CD_3OD.

NO.	δ_H (J in Hz)	δ_C	NO.	δ_H (J in Hz)	δ_C
1		134.3	5'		155.0
2	7.46 (d, 8.7)	133.2	6'	6.44 (s)	104.8
3	6.85 (d, 8.7)	131.0	1''		126.5
4		158.1	2''	7.19 (d, 8.7)	116.2
5	6.85 (d, 8.7)	131.0	3''	6.80 (d, 8.7)	115.5
6	7.46 (d, 8.7)	133.2	4''		157.2
1'		134.3	5''	6.80 (d, 8.7)	115.5
2'		140.8	6''	7.19 (d, 8.7)	116.2
3'		149.3	2'OCH_3	3.38 (s)	60.7
4'		118.7	5'OCH_3	3.68 (s)	56.4

Table S7. ^{1}H NMR and ^{13}C NMR data of compounds SF9[8] in $CDCl_3$.

NO.	δ_H (J in Hz)	δ_C	NO.	δ_H (J in Hz)	δ_C
1	1.93, 1.31 (m)	31.3	15	4.92 (m)	70.8
2	2.02, 1.64 (m)	35.3	16	2.10, 1.70 (m)	36.0
3	3.76 (m)	70.1	17	1.85 (m)	51.4
4	2.34 (m)	41.7	18	1.03 (s)	19.8
5		168.4	19	1.09 (s)	19.1
6	5.34 (d, 2.2)	127.9	20	2.15 (m)	38.6
7		190.4	21	1.06 (d, 6.1)	21.7
8		126.3	22	5.27 (dd, 15.2, 7.7)	134.8
9	2.60 (m)	47.3	23	5.21 (dd, 15.3, 7.7)	132.2
10		38.6	24	1.86 (m)	43.1
11	1.73 (m)	19.4	25	1.46 (m)	33.2
12	2.10, 1.29 (m)	35.6	26	0.82 (d, 6.0)	20.0
13		44.9	27	0.83 (d, 6.1)	20.2
14		168.8	28	0.92 (d, 6.0)	17.8

SF9 possessed negative optical rotation value ($[\alpha]_D^{20}$ -216.4, c 0.1, MeOH). On the basis of chemical shift values of C-15/H-15 (Table S7) and C-3/H-3, the relative configuration was identified as 3*β*, 15-*α*. [8].

References

1. Takagi, M.; Motohashi, K.; Shin-ya, K. Isolation of 2 New Metabolites, JBIR-74 and JBIR-75, from the Sponge-Derived Aspergillus Sp. fS14. *J Antibiot* **2010**, *63*, 393–395, doi:10.1038/ja.2010.58.
2. Lopez, J.A.V.; Nogawa, T.; Futamura, Y.; Aono, H.; Hashizume, D.; Osada, H. N-Acetyl-α-Hydroxy-β-Oxotryptamine, a Racemic Natural Product Isolated from Streptomyces Sp. 80H647. *J Antibiot* **2021**, *74*, 477–479, doi:10.1038/s41429-021-00420-7.
3. Ren, L.; Wang, Y.-Z.; Zhang, W.; Zhou, R.; Zhao, M.; Tang, Z.-S.; Sun, J.; Zhang, D.-B. Triculata A, a Novel Compound from Tricyrtis Maculata (D. Don) J. F. Macbr. with Biological Properties. *Nat Prod Res* **2021**, *35*, 3729–3737, doi:10.1080/14786419.2020.1736059.
4. Pedras, M.S.; Khan, A.Q. Biotransformation of the Phytoalexin Camalexin by the Phytopathogen Rhizoctonia Solani. *Phytochemistry* **2000**, *53*, 59–69, doi:10.1016/s0031-9422(99)00479-3.
5. Pei S. L.; Chen L.; Xu J. L.; Shao C. L. Secondary Metabolites and Their Biological Activities of Two Actinomycetes Streptomyces Coelicoflavus and Nocardiopsis Dassonvillei Associated with Ascidians Styela Clava and Botryllus Schlosseri. *Chinese Journal of Marine Drugs* **2017**, *36*, 55–60, doi:10.13400/j.cnki.cjmd.2017.02.009.

6. Jin, H. G.; Liu, K. Y.; Qu, W. H.; Li, T. J.; Liao, L.; Yu, J. M. Isolation and Structure Identification of Chemical Constituents from the Fruits of Akebiae Quinata. *Natural Product Research and Development* **2019**, *31*, 2077–2081, 2064, doi:10.16333/j.1001-6880.2019.12.009.
7. Marchelli, R.; Vining, L.C. Terphenyllin, a Novel p-Terphenyl Metabolite from Aspergillus Candidus. *J Antibiot (Tokyo)* **1975**, *28*, 328–331, doi:10.7164/antibiotics.28.328.
8. Wang, F.; Fang, Y.; Zhang, M.; Lin, A.; Zhu, T.; Gu, Q.; Zhu, W. Six New Ergosterols from the Marine-Derived Fungus *Rhizopus* Sp. *Steroids* **2008**, *73*, 19–26, doi:10.1016/j.steroids.2007.08.008.
